# Survivin as a Preferential Target for Cancer Therapy

**DOI:** 10.3390/ijms15022494

**Published:** 2014-02-13

**Authors:** Mahsa Mobahat, Aru Narendran, Karl Riabowol

**Affiliations:** 1 Department of Biochemistry & Molecular Biology, Faculty of Medicine, University of Calgary, 3330 Hospital Dr. NW., Calgary, AB T2N 4N1, Canada; E-Mail: mmobahat@ucalgary.ca; 2 Department of Pediatrics, Faculty of Medicine, University of Calgary, 3330 Hospital Dr. NW., Calgary, AB T2N 4N1, Canada; E-Mail: a.narendran@ucalgary.ca; 3 Department of Oncology, Faculty of Medicine, University of Calgary, 3330 Hospital Dr. NW., Calgary, AB T2N 4N1, Canada

**Keywords:** apoptosis, survivin, inhibitor of apoptosis (IAP), signaling, cancer therapy

## Abstract

Cancer is typically a consequence of imbalance between cell death and proliferation in a way favorable to cell proliferation and survival. Most conventional cancer therapies are based on targeting rapidly growing cancerous cells to block growth or enhance cell death, thereby, restoring the balance between these processes. In many instances, malignancies that develop resistance to current treatment modalities, such as chemotherapy, immunotherapy, and radiotherapy often present the greatest challenge in subsequent management of the patient. Studies have shown that under normal circumstances, cells utilize different death mechanisms, such as apoptosis (programmed cell death), autophagy, mitotic catastrophe, and necrosis to maintain homeostasis and physiological integrity of the organism, but these processes often appear to be altered in cancer. Thus, in recent years developing various strategies for administration of cytotoxic chemotherapeutics in combination with apoptosis-sensitizing reagents is receiving more emphasis. Here, we review the properties of the anti-apoptotic protein, survivin, a member of the inhibitor of apoptosis protein (IAP) family and the clinical feasibility and anti-cancer potential of drugs targeting this protein. We also discuss some key points and concerns that should be taken into consideration while developing drugs that target apoptotic proteins, such as survivin.

## 1. Apoptosis, a Developmental and Defense Mechanism

In addition to playing critical roles in development, apoptosis is a natural defense mechanism to eliminate damaged and/or unhealthy cells that arise in many ways. In mammalian systems it is known to occur through two distinct pathways: the extrinsic pathway utilizing ligands and cell surface death receptors and the intrinsic pathway that includes the mitochondrion as a key component. Most conventional chemotherapies and radiotherapies target the intrinsic pathway. Choosing apoptosis and the intrinsic mechanism of programmed cell death as a target for cancer treatment has both merits and disadvantages for new drug development since some degree of apoptosis is necessary for organ function, but excess apoptosis, particularly in non-renewing cell types can obviously have detrimental consequences. Therefore, balance is critical and a detailed understanding of the different apoptosis pathways and their regulators, the signals triggering the activation and repression of apoptosis and, perhaps most importantly the consequences of altering apoptosis in tumors and surrounding cells, should provide novel targets for more efficient cancer therapeutics.

The ability to activate or up-regulate pro-apoptotic regulators and/or down-regulate or silence anti-apoptotic mechanisms has been facilitated by our past and current and understanding of the natural mediators of apoptosis. However, a major challenge remains in designing approaches that target efficiently and result in effective tumor cell killing but minimal effects on normal cells. Therefore, further understanding of different apoptosis pathways and their regulators, the signals triggering the activation and repression of apoptosis and importantly the consequences of apoptotic treatments in tumor microenvironments should provide novel and more efficient avenues for the development of efficacious cancer therapeutics. The major pathway that we will focus upon in this review is the mitochondria-mediated apoptosis (intrinsic) pathway.

### 1.1. Intrinsic Apoptosis Pathway (Mitochondrial Pathway)

The intrinsic apoptosis pathway is activated by various death-inducing stimuli, such as DNA damage signals that are often generated by chemotherapy and radiation. Several key regulators of this pathway belong to the Bcl-2 family of molecules, consisting of pro-apoptotic and anti-apoptotic proteins containing Bcl-2 homology domains (BH domains). The pro-apoptotic regulators have been subdivided into two groups: the Bax subgroup that includes Bax, Bak, and Bok proteins, having three BH domains (BH1, BH2, and BH3) and the BH3-only group that includes Bid, Bad, Bik, Bim, Noxa, Puma, and Bmf.

The anti-apoptotic group of proteins is comprised of Bcl-2, Bcl-X_L_, Bcl-w, Bfl-1, and Mcl-1 proteins, possessing four BH domains (BH1, BH2, BH3, BH4) [1–3]. One major determinant of whether a cell enters an apoptotic phase in response to cellular insults that activate upstream signaling pathways is the final stoichometry between pro- and anti-apoptotic proteins containing BH domains with anti-apoptotic members binding to, and neutralizing pro-apoptotic members. If the balance shifts past a critical value in favor of pro-apoptotic members, these proteins are able to bind at the mitochondrial membrane to decrease its integrity, resulting in membrane depolarization and release of mitochondrial contents into the cytoplasm. Cytochrome C, in particular, subsequently plays a central role in activating the apoptosome, triggering activation of the effector cysteine-dependent aspartyl-specific proteases (caspases) which then contribute to further the apoptotic cascade by digesting a broad range of cellular substrates [4].

In addition to the BH family, another major group of proteins called the inhibitor of apoptosis (IAP) proteins play a powerful role in regulating the intrinsic pathway of programmed cell death. Particularly well-characterized members of this family include XIAP (X-chromosome-linked IAP), cIAP1 (cellular IAP1), cIAP2 (cellular IAP2), Apollon, ML-IAP (melanoma IAP), survivin, IAP-like protein 2 (ILP2), and NAIP (neuronal apoptosis inhibitory protein) [5,6]. As some of these regulatory proteins have been implicated in the survival of tumor cells in response to cytotoxic stress induced by cancer therapies, they have provided an attractive target for cancer drug development that we discuss further, below.

### 1.2. The Structure and Function of IAP Proteins

**Figure 1.**
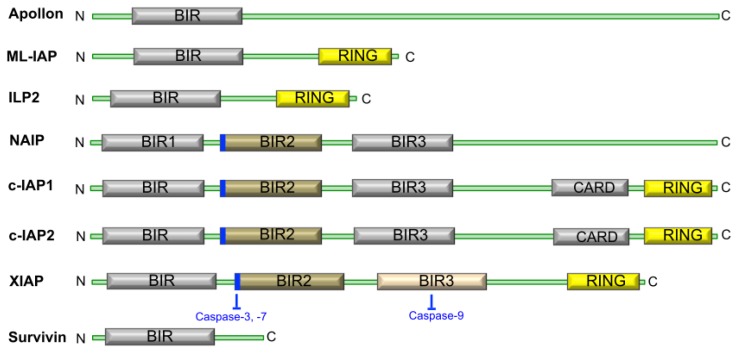
Inhibitor of Apoptosis Protein (IAP) structure in mammals. The IAP family of proteins consists of eight proteins including Apollon, ML-IAP (melanoma IAP)/Livin, ILP2 (IAP-like protein-2), NAIP (neuronal apoptosis-inhibitory protein), c-IAP1, c-IAP2, XIAP (X-linked IAP), and survivin. A conserved linker peptide that precedes the BIR2 (baculoviral IAP repeat-2) domain of XIAP, c-IAP1, c-IAP2, or NAIP (shown in blue) is responsible for inhibiting caspases-3 and -7. In XIAP, the BIR3 domain inhibits caspase-9.

The IAP family of proteins contain one to three baculovirus IAP repeat or BIR domains (Figure 1), which are essential to their anti-apoptotic function. Many of the cellular IAPs also possess a really interesting new gene (RING) domain at their *C*-termini, and some also contain a caspase recruitment domain or CARD [7].

Currently, controversy exists over the precise mechanism by which the IAPs inhibit apoptosis. However, there is evidence to suggest that XIAPs can directly bind caspases 3, 7, and 9 and inhibit their function [8] using the short regions highlighted in Figure 1. It has also been reported that this inhibition occurs through the ability of IAPs to promote ubiquitination and therefore proteasomal degradation of the caspase in question [9]. The IAP regulatory protein SMAC (Second Mitochondria-derived Activator of Caspases, or the mouse homolog DIABLO) is localized to the inter-membrane space of mitochondria under normal, non-apoptotic conditions and is released, together with cytochrome C, in response to apoptotic stimuli that affect the ratio of pro-and anti-apoptotic BH family members. As a consequence of pro-apoptotic BH family members forming dimers and permeabilizing the mitochondrion, SMAC is released and binds IAP proteins through an IAP binding motif. This displaces caspase-9 from the complex, resulting in disruption of the inhibitory activity of IAPs on caspases [10–14] and, thus, SMAC serves as an inhibitor of the inhibitors of apoptosis. The other three IAP proteins, c-IAP1, c-IAP2, and ML-IAP have also been reported to bind SMAC, thus, segregating SMAC from XIAP allowing it to remain active as an inhibitor. Finally, caspase-independent cell functions have also been identified for IAPs where studies in human, yeast, and *C. elegans* systems provide evidence for roles of IAPs in regulating cell division, particularly during cytokinesis [15–19].

### 1.3. IAP Proteins and Cancer

Undoubtedly, suppression of apoptosis is a hallmark of the majority cancers that typically become genetically unstable, which normally triggers an apoptotic response in non-cancerous cells [20]. Consistent with this idea, increased levels of different members of the IAP family have been reported in many cancer types [21–23] and over-expression of IAP proteins has been reported to enhance resistance to apoptotic stimuli in many malignancies [24–26]. Thus, a concerted effort has been mounted to further examine the precise role of IAPs in tumor development and to explore their potential as targets for cancer therapy. In this family of proteins, survivin has taken a center stage, due to its markedly specific expression in cancer cells.

### 1.4. Survivin, a Crucial IAP Target in Cancer Therapy

Having 142 amino acid residues, survivin (also called Baculoviral Inhibitor of Apoptosis Protein Repeat-Containing 5 or BIRC5) is the smallest IAP, with the unique characteristic of having a single BIR domain (Figure 1). Various studies have suggested different mechanisms by which survivin levels might be regulated. A summary of the studies and processes proposed to control survivin expression, protein levels or activity is displayed in Table 1.

It has been believed for a long time that the survivin protein is rarely expressed in healthy adult tissues despite its expression during fetal development. Although recent reports suggest roles for survivin in normal cells [44] including T-cells [45], hematopoietic progenitor cells [46], vascular endothelial cells [47], liver cells [48], gastrointestinal tract mucosa [49], erythroid cells [50], and polymorphonuclear cells [51], survivin expression is significantly higher in transformed cells suggesting a pathological role for the protein [52]. For example, clinical studies revealed that 60% of biopsy samples from neuroblastoma (stage3–4) patients [53], 53% of colon cancer [54], 50% of high-grade lymphomas [55] and 35% of gastric cancer [56] are survivin positive. The only exception reported is low-grade lymphoma in which no expression of survivin was observed [55].

### 1.5. Function of Survivin

Survivin was shown to inhibit apoptosis both *in vitro* and *in vivo* [57,58], perhaps via interactions with multiple regulators of both intrinsic and extrinsic apoptosis pathways. Survivin is negatively regulated by p53, both at the mRNA and protein levels [59]. In addition, over-expression of survivin rescues a p53-induced apoptosis phenotype [59]. It has been shown that survivin inhibits Fas (CD95)-mediated apoptosis by supporting caspase3/p21 formation as a result of interaction with cdk4 [60]. In addition, survivin was shown to suppress the cell death induced by TRAIL [61] and Bax [62]. Regarding caspase-dependent roles of survivin, various, and often controversial, data have been reported. Although many studies report evidence for interactions between survivin and initiator and effector caspases [38,62–65], some suggest that this interaction does not result in caspase inactivation [66]. These conflicting data suggest that survivin may inhibit apoptosis by caspase-independent mechanisms under certain conditions.

### 1.6. Survivin as a Nodal Protein

**Figure 2.**
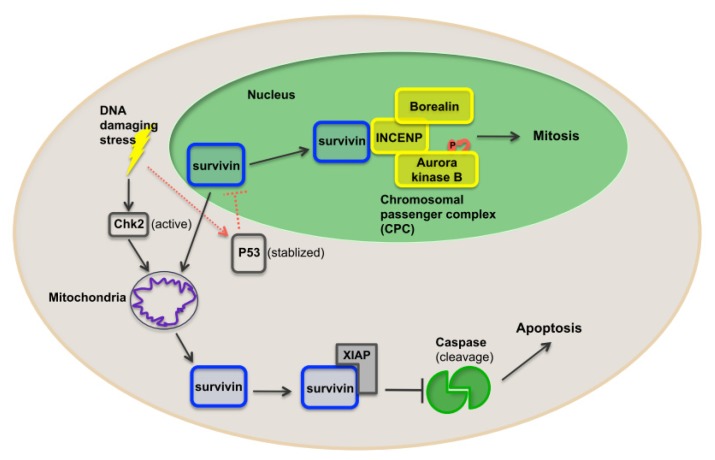
Pathways through which survivin can favor tumor cell development. Survivin is a component of the chromosome passenger complex (CPC) and a key regulator of chromosome segregation and cytokinesis. The association of survivin with two other components of the CPC complex, INCENP (inner centromere protein antigen) and borealin, regulate the localization of the aurora kinase B enzymatic component, to kinetochores. Aurora kinase B undergoes auto-phosphorylation upon recruitment to the CPC complex, promoting correct chromosome alignment, segregation and cytokinesis during mitosis. In addition, DNA damage-induced activation of checkpoint kinase 2 (Chk2) results in rapid release of survivin from mitochondria, inhibiting cell death and promoting tumor cell survival. DNA damage also stabilizes wild-type p53, repressing the transcription of survivin and helping to balance this pathway.

Due to its role in many different cellular actions and signaling pathways, survivin has been described as a nodal protein (Figure 2). In addition to a role in suppressing apoptosis, survivin is also a mitotic regulator involved in various cell division processes. One of the more remarkable functions of survivin revolves around its localization at the mitotic apparatus [67]. Survivin is a component of the chromosomal passenger complex (CPC) and thereby functions as a key regulator of chromosomal segregation and cytokinesis [68]. CPC localizes to centromeres and subsequently associates with central spindle midzones and the midbody. The association of survivin with two other components of the CPC complex, INCENP (inner centromere protein antigens), and Borealin, regulates the localization of the enzymatic component, Aurora kinase B, to kinetochores [68] and, subsequently, facilitates chromosome alignment, segregation and cytokinesis during mitosis. In addition, it has been shown that DNA damage-induced activation of the checkpoint kinase 2 (CHK2) results in rapid release of survivin from the mitochondria and consequently inhibition of cell death, helping to promote tumor cell survival [69]. DNA damage stimuli also stabilize p53, which in turn can repress the transcription of survivin and help balance the degree to which activation of CHK2 promotes survivin release and activation of caspases [29]. In addition, survivin associates with microtubules contributing to spindle formation [70]. Microtubule-assembled survivin is phosphorylated at mitosis upon association with CDK1. As a consequence, survivin is stabilized during mitosis, repressing cell death in mitotic cells [38].

### 1.7. Survivin Induces Chemoresistence

Early data indicated that survivin renders resistance to chemotherapy agents in tumor cells [71]. It this study, HeLa cells expressing survivin from an adenoviral vector showed resistance to apoptosis induced by the microtubule stabilizer taxol (also called paclitaxel). Subsequently, it was shown that forced expression of wild-type survivin suppressed cytotoxicity induced by taxol in human ovarian [72] and prostate cancer cell lines [73] and taxol-induced cell cycle arrest leads to increase in the expression of survivin [74].

As noted above, significantly high expression of survivin has been reported in many cancers including lung, colon, breast, prostate, bladder, breast, laryngeal, uterine, hepatocellular, soft tissue sarcomas, renal, pancreas, high-grade lymphomas, neuroblastomas, and gastric cancers, as well as in hematologic cancers, such as myelodysplastic syndrome and acute leukemias [53–56,75]. High levels of survivin expression have also been observed in colorectal cancer cell lines resistant to the tumor necrosis factor-related apoptosis inducing ligand (TRAIL) [76], as well as in prostate cancer [77] and thyroid cancer cell lines resistant to cisplatin [78]. Survivin was also suggested to induce resistance to flutamide anti-androgen therapy in prostate cancer cells [79]. These data suggest the exciting possibility that more efficient chemotherapeutic approaches can be developed in combination with inhibitors of survivin.

### 1.8. Survivin Induces Radioresistence

There is also evidence that survivin suppresses radiation-induced cytotoxicity. In experiments using pancreatic cancer cell lines, survivin mRNA expression and radiosensitivity showed an inverse relationship [80]. In the same set of experiments, survivin expression was increased upon treatment with a sublethal dose of X-irradiation. This inverse relationship between survivin expression and radiosensitivity has also been observed in colorectal cancer, glioblastoma, and melanoma cell lines [81,82]. Taken together, these findings suggest that the inhibition of survivin has the potential to also enhance the effects of radiotherapy in cancer patients.

### 1.9. Survivin as a Cancer Diagnostic Marker

As mentioned above, expression of survivin is substantially different in tumor cells compared to normal cells. In the past few years survivin has emerged as a potential early predictor of malignancies. In a study on oral cancerous lesions, 33% of precancerous (without malignant progression) and 94% of precancerous lesions that evolved into full-blown squamous cell carcinomas showed expression of survivin [83]. Interestingly, tumors that transformed from these precancerous lesions preserved 100% of their survivin positivity. These promising reports suggest that survivin could serve as a marker for the diagnosis of malignancies at early stages.

### 1.10. Survivin as a Cancer Prognostic Marker

Survivin’s over-expression at the mRNA and proteins levels appears to correspond with higher malignant grades and reduced survival rates in different cancers such as hepatocellular carcinoma [84], esophageal cancer [85], glioblastoma [86], lung cancer [87], B-cell non-Hodgkin’s lymphoma, and breast cancer [88,89] patients. In a study of 55 patients with hepatocellular carcinoma after hepatectomy, the recurrence rate was significantly higher in those with survivin-positive tumors. Moreover, the 1- and 3-year survival rates for survivin-positive patients were substantially lower than for survivin-negative patients [84]. Likewise, reports in many other cancer patients are in agreement with the above-suggested correlation between survival and recurrance rates with survivin levels [89]. Interestingly, few reports suggest the localization of survivin as a prognostic biomarker. A report on patients with glioblastoma indicated that while cytoplasmic survivin levels did not have any effect on prognosis, nuclear survivin localization correlated with significantly lower survival rate than that of patients with low nuclear survivin levels [86]. On the other hand, nuclear survivin level did not show any correlation with survival in non small cell lung carcinoma (NSCLC) patients [90]. Thus, using survivin as a general prognostic marker needs further verification, however, so far findings in several cancers show clear promise for this potential biomarker.

### 1.11. Therapeutic Targeting of Survivin

**Figure 3.**
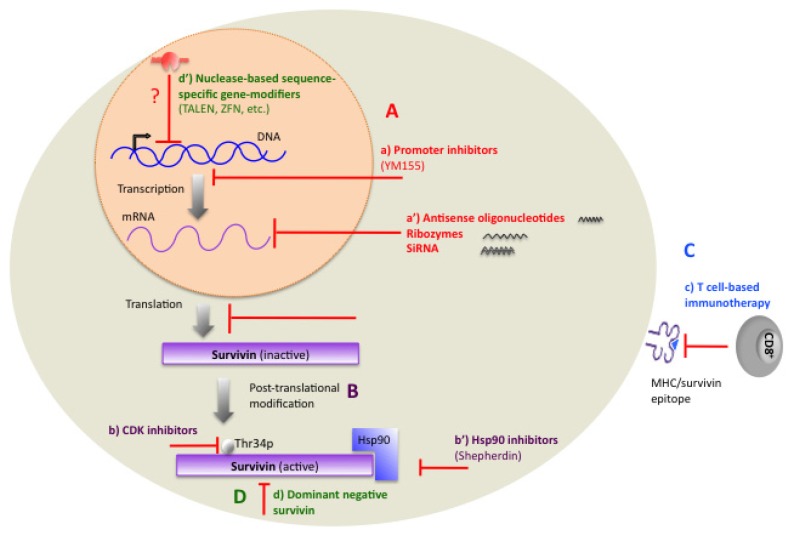
Therapeutic targeting of survivin. Major classes of therapeutic agents targeting survivin are depicted in the figure. Such treatments include drugs that (**A**) function at the transcription level and inhibit the transcription of survivin (such as promoter inhibitors (**a**), Antisense oligonucleotides, Ribozymes, and SiRNA (**a**′), (**B**) inhibit survivin at post-translational level (such as CDK inhibitors (**b**) and Hsp90 inhibitors (**b**′)), (**C**) include vaccines that are based on cytotoxic activities of CD8^+^ T lymphocytes against specific survivin epitopes (**c**) or (**D**) gene therapy methods including transfecting with dominant negative mutants (**d**) which encode proteins that suppress survivin’s function. Within this class, we propose that use of the breakthrough technology of nuclease-based genome-editing tools (**d**′), may show promising results once designed and applied against survivin in pre-clinical trials.

Significant efforts have been focused on developing strategies to use survivin as a target for therapeutics in cancer. Developing drugs that target survivin might initially seem difficult because survivin is not an enzyme nor it is a cell surface protein. However, considerable progress has been made to achieve optimal efficiency in suppressing survivin. These strategies have been conducted using a variety of approaches as outlined in Figure 3. Among these the most promising therapies are discussed below.

## 2. Transcriptional Inhibitors

Antisense oligonucleotides (AO) are short, single stranded RNA or DNA sequences that are complimentary to a specific RNA strand that act by hybridization to the target mRNA strand to suppress the expression of the particular gene. The first attempt for AO therapy targeting survivin triggered apoptosis in human melanoma cell lines [91]. Successively, subsequent studies using AOs against survivin confirmed that small chemically synthesized oligonucleotides or expression vectors encoding them are capable of specifically inhibiting survivin either at mRNA or protein levels. This led to decreased cell proliferation and increased caspase-dependent apoptosis in different tumor (lung, sarcomas, lymphomas, thyroid, head, and neck) cell lines [92–96]. Furthermore, down-regulation of survivin via AO was shown to enhance sensitivity to cytotoxic agents such as TRAIL [61,63], cisplatin [93], taxol [97], imatinib [98], etoposide [93,99,100], as well as to cytotoxicity induced by radiation therapy [101]. Moreover, treatment with survivin AO resulted in inhibition of tumor growth in murine models [102]. Although these reports using AO-therapy are very promising, caveats worth mentioning are the inefficient neutralization of the target mRNA and *in vivo* instability. Despite these limitations, the first AO drug, LY2181308, has entered phase II of clinical trials.

Ribozymes are small RNA molecules that cleave target RNA by their endonucleolytic activity. In different studies, transfection of human melanoma cell lines with ribozymes led to reduced expression of survivin protein and augmented caspase-9-dependent cell death and sensitivity to *cis*-platinum, topotecan, and radiation [81,103,104]. The potential caveat to use of ribozyme administration is that ribozymes degrade easily and cause aberrant cell trafficking. However, strategies to overcome these limitations and increase the efficacy of these drugs for use *in vivo* are being developed. Thus far, none of the drugs of this family have entered clinical trials.

Small interfering RNAs (siRNAs) are short, double-stranded RNAs that inhibit gene expression. SiRNAs are often more effective than other antisense approaches, rendering them better candidates for clinical treatments because lower concentrations of them is needed for each treatment resulting in less side effects compared to other techniques. The first RNAi-mediated inhibition of survivin resulted in delayed mitosis, misaligned chromosomes and accumulation in prometaphase in Hela cells [105,106]. Further, in preclinical studies that attempted to utilize RNAi technology (either plasmid vectors encoding short hairpin RNAs or chemically synthesized siRNAs) to target survivin resulted in decreased survivin expression, enhanced cell death, reduced cell proliferation and inhibited tumor growth [106–115]. Suppression of survivin by RNAi also enhanced the sensitivity of cancer cell lines to multiple treatments such as vincristine [116], 17-allylamino-17-demethoxygeldanamycin [108], doxorubicin [113,115], APO2L/TRAIL [109], TNF-alpha [113], and, as with AO and ribozymes, to radiation [113,114].

## 3. Small-Molecule Antagonists

### 3.1. Hsp90 Inhibitors

It is believed that interaction of survivin with the molecular chaperone Hsp90 stabilizes the survivin protein [33]. Shepherdin is a cell-permeable antagonist of the Hsp90-survivin complex and acts through counteracting the binding of these two proteins. In addition, shepherdin was shown to destabilize many Hsp90 client proteins such as Akt, telomerase and CDK6. Moreover, shepherdin induces caspase-dependent and caspase-independent cell death in various cancer cells both *in vitro* and *in vivo* and suppresses growth in breast and prostate cancers in xenograft models [117]. Treatment with shepherdin has not shown any effect on normal cells except for a weak tocxicity on normal fibroblasts upon increase in drug dose. Shepherdin is currently in the pre-clinical phase.

### 3.2. Cyclin-Dependent Kinase (CDK) Inhibitors

CDK inhibitors can counteract mitotic phosphorylation of survivin on Thr^34^ and thereby lead to the destruction of survivin and its function. Flavopiridol and purvalanol A are members of this group of inhibitors. Strikingly, treatment with CDK inhibitors *in vivo*, results in loss of survivin expression, escape from taxol-mediated mitotic block, mitochondrial-dependent and p53 independent apoptosis, as well as robust antitumor activity [74]. Interestingly, inhibition of survivin phosphorylation was reported to be the potential underlying mechanism that some of the CDK inhibitors act by to induce taxol-induced apoptosis in HeLa cells [118].

### 3.3. Promoter Inhibitors

The imidazolium-based compound YM155 has, so far, emerged as the most important in this group of low molecular weight antagonists. YM155 specifically inhibits survivin gene expression by suppressing promoter activity and subsequent gene expression in cervical, colon, lung, and other cancer cells. It also diminishes proliferation and tumor growth in NSCLC, lymphoma and prostate cancer xenografts [119]. YM155 is currently in phase II clinical trial in patients with refractory diffuse large B-cell lymphoma. At the same time, a phase I clinical trial is evaluating the feasibility of combination-therapy of YM155 with docetaxel and prednisone for patients with hormone refractory prostate cancer.

### 3.4. Other Low Molecular Weight Antagonists

Another low molecular weight antagonist of survivin is terameprocol, also called EM-1421. The basic function of this drug is believed to be the inhibition of Sp1 regulated proteins such as survivin and Cdc2, which subsequently inhibits tumor progression [120,121]. Terameprocol is in phase I clinical trials for refractory solid tumors and lymphoma and in a phase II clinical trial for leukemia.

## 4. Immunotherapy

The recognition of specific tumor-associated antigens by T lymphocytes forms the basis for cancer immunotherapy. Earlier reports using immunotherapy for targeting survivin, revealed that cytotoxic CD8^+^ T lymphocytes have significant cytolytic activity against specific survivin epitopes *in vitro* and *in vivo*. Interestingly, a strong rationale for developing survivin-based anti-cancer vaccines emerged when the above findings were confirmed in mouse models of lymphoma and pancreatic cancer by the identification of cytotoxic CD8^+^ T lymphocytes that were responsive to some of survivin’s epitopes. These vaccines were able to induce tumor suppression in multiple animal cancer models including lung [122], lymphoma, neuroblastoma [123], and pancreatic and hormone refractory prostate cancers [124]. Importantly, no significant toxicity was observed in studies that have been reported to date. Promising results from initial survivin-based vaccines against different malignancies have led to many ongoing investigations focusing on finding the epitopes that generate the strongest immunodominant, immunoprevalent T-cell reaction against survivin. Many of these drugs are currently in phase I or phase II clinical trials.

## 5. Gene Therapy

### 5.1. Dominant-Negative Mutants

The use of gene therapy was one of the earliest strategies used to inhibit survivin activity. In an initial study, induction of apoptosis was observed upon transfecting melanoma cells with survivin dominant-negative expression constructs [91]. Subsequent studies reported that transfection with dominant-negative mutants of survivin led to suppression of growth and increased apoptosis in gastric cancer cell lines [125], and to suppression of tumor growth in breast cancer [126] and thymic lymphoma [102] animal models. In addition, survivin dominant-negative therapy increased sensitivity to cisplatin and 5-fluorouracil [125]. Interestingly, mice expressing dominant-negative survivin showed decreased probabilities of developing tumors or exhibiting tumor-associated angiogenesis [125]. The results from experiments with other dominant-negative mutants of survivin agreed with those of the first mutation used. They also showed that treating different cancer cell lines [126], as well as melanoma [127] and breast cancer [126,128] xenografts with dominant-negative mutants of survivin resulted in suppression of tumor growth and angiogenesis [128] and augmentation of apoptotic responses. Intriguingly, *in vitro* data showed no effect of these mutants on the growth of normal endothelial, fibroblast or smooth muscle cells [126].

### 5.2. ZFN, TALEN, and CRISPR/Cas-Based Methods, as Potential Methods for Targeting the Survivin Gene in Tumor Cells

In the last decade, the advent of novel “genome editing” techniques, which are based on site-specific-directed nucleases, has raised hopes for enhanced feasibility of targeting the main causes of many diseases. The potential for therapeutic application of Zinc-finger nuclease (ZFNs), transcription activator-like effector nucleases (TALENs) or clustered regulatory interspaced short palindromic repeat (CRISPR/Cas-based) RNA-guided DNA endonucleases [129–131] has enabled a paradigm-shift compared to conventional gene therapy methods.

In general, such genome modifying tools are composed of two main domains: (1) a designable and sequence-specific DNA-binding domain; and (2) a nuclease domain, which modifies the specific sequence of interest by leaving double-strand breaks behind, in the locus. The damage, in the form of double-strand breaks, will further undergo error-prone mechanisms of DNA repair, such as non-homologous end joining (NHEJ) or homology-directed repair (HR), thereby, impacting the locus by an extended variety of mutations such as gene disruption, insertion, deletion, inversion, addition or correction.

To date, conventional genome engineering techniques have been limited by several factors such as low efficiency, low specificity and labor-intensity. However, the emergence of the novel strategies in disease treatment, such as ZFN, TALEN, or CRISPR/Cas-based RNA-guided DNA endonucleases presents promise to overcome such limitations in future. This new technology, with further high-throughput validations of stringency and specificity, could potentially revolutionize cancer treatment. Performing personalized “gene-manipulation” therapies for oncogenes in cancer patients would be one possibility following from the current efforts to optimize targeted nucleases. For example, designing ZFN or TALEN to target the survivin gene in cancer cells, would trigger cell death specifically in transformed cells, while having minimal effect on normal cells. As with other gene therapy approaches, a major limitation is the likely requirement to target all, or a great majority of cancer cells for the therapy to be durable so the development of more efficient vectors may be the step limiting effectiveness of this approach.

## 6. Potential Caveats and Alternative Approaches

Despite the considerable potential for altering survivin levels as a method for helping to selectively eliminate cancer cells, there are some cautions that have been overlooked to various degrees. For example, the heterogeneous microenvironment of tumors has strong influences on tumor progression and also on the effectiveness and/or toxicity of tumor suppressor therapeutics. Indeed, apoptotic cells can induce immunosuppressive and anti-inflammatory effects on their neighboring cells. Specifically, repression of pro-inflammatory factors such as TNF-α, IL-1β, IL-8, and IL-12, as well as production of TGF-β (as anti-inflammatory mediator), are induced through contact with apoptotic cells [132–135]. Cells juxtaposed with apoptotic cells also generate differentiation and growth signals. These signals include growth and even pro-angiogenesis factors, such as VEGF and hepatocyte growth factor [136–138]. In addition, FKN, a survival and proliferative factor, is secreted from apoptotic cells [139]. Furthermore, release of pleiotropic anti-inflammatory factors such as TGF-β, lactoferrin and IL-10 by apoptotic cells can result in growth and differentiation in neighboring cells, favoring overall tumor progression. This phenomenon is best described *in vivo* in *Drosophila* and is referred to as “compensatory proliferation”. In this case, apoptotic cells stimulate the neighboring surviving cells to proliferate. Considering the possibilities of any type of “compensatory proliferation” in response to apoptosis-induction therapies would be crucial because it directly influences the outcome of the treatment in favor of either tumor regression or growth. However, this is obviously not a caution unique to survivin-based apoptotic therapeutics and indeed it is an important issue that should be taken into consideration in pre-clinical stages of development of any cytotoxic drug that activates apoptosis.

## 7. Conclusions

Developing strategies that cause cell death specifically in tumors is a central theme in cancer treatment. However, after many years of considerable efforts, fundamental challenges such as tumor specificity, sustainability and toxicity of treatments and durability of patient response remain. Recently, an additional concern has arisen regarding the potential of cytotoxic cancer therapeutics to have growth-promoting effects on neighboring normal cells. The initial studies that noted the idea of tumor repopulation after cytotoxic treatments originated more than 40 years ago [140,141], where tumor cell repopulation was observed after exposing tumors to radiotherapy. The mechanisms underlying increased proliferation after inducing cell-death in tumor cells have been elusive for many years. In this context, a recent study by Huang *et al*. [142] showed that caspase-3 activation in response to radiotherapy stimulates tumor repopulation. This has generated concerns with respect to some of the deleterious consequences of cytotoxic agents under certain circumstances. Thus far, adjuvant use of inhibitors of repopulation-stimulating pathways, along with cell-death-inducing therapies, is one possible approach to overcome this potential concern.

The differential expression of survivin in malignant *versus* normal cells is a strong rationale for development of survivin-based cancer therapeutics, from which many ongoing trials have ensued using a variety of methods to alter survivin or target it in cancer cells. One method, taking advantage of tumor-specific promoters like that of survivin, may allow future studies to potentially direct the expression of other relevant apoptosis-inducing genes or apoptosis-inhibiting mutants in tumors. For example, in a recent study [143], the survivin promoter was used to direct expression of other apoptotic genes selectively in tumor cells. Results from these and other approaches support the idea that the survivin gene and promoter might have continued utility for developing novel agents to target and attack tumors of various types.

## Figures and Tables

**Table 1.** Summary of the main pathways through which survivin is regulated. Survivin levels and localization can be regulated by changes in transcription, physical association with chaperones, altering proteosomal degradation, and by other post-translational mechanisms such as phosphorylation and acetylation of key amino acid residues.

Type of regulation	Reported interaction	Cell line(s)	References
*Transcriptional regulation*			
Sp1/Sp3	As transcription factors, positively regulate survivin promoter activity	Human HeLa cervical adenocarcinoma cells	[27]
RB/E2F	E2F activators (such as E2F1, E2F2 and E2F3) positively and RB negatively regulates survivin promoter activity	Rat embryo fibroblasts, human WI-38 fibroblasts, normal human melanocytes	[28,29]
p53	Competes with the binding of E2F activators to survivin promoter, hence inhibits survivin transcription	Normal human melanocytes	[29]
NICD (Notch-intracellular domain)	Translocation of NICD to the nucleus activates survivin promoter	Colon cancer cells and human non-small cell lung cancer cells	[30,31]
IGF-1	Enhances translation of survivin	Human DU145 prostate cancer cell line	[32]
*Regulation of survivin protein stability*			
HSP90	Stabilizes survivin through physical association	HeLa cervical carcinoma cells	[33]
EGF and EGFR pathways such ERK and AKT signaling pathways	Inhibits poly-ubiquitination and thus degradation of survivin	Mouse MIN6 and rat INS-1 pancreatic β cells, MCF-7 breast cancer cells	[34–36]
VEGF (vascular endothelial growth factor)	Increased VEGF enhances survivin protein levels through activating PI3K/AKT pathway	Neuroblastoma cells	[37]
*Post-translational regulation of survivin*			
Phosphorylation on threonine 34	Thr34-phosphorylated survivin binds caspase-9 and inhibits apoptosis	Human oral squamous adenocarcinoma cells, oral fibrosis, HeLa cells	[38–40]
Dephosphorylation on serine20	Ser20-dephosphorylation translocates survivin from mitochondria to cytoplasm to inhibit caspase cleavage	Insulinoma INS-1 cells	[41,42]
Acetylation on lysine 129	CREB-binding protein acetylates survivin on lysine 129 to increase survivin nuclear accumulation, decreasing cell survival	HEK293T, HeLa cells and MCF-7 breast cancer cells	[43]

## References

[1] Reed J.C. (1997). Bcl-2 family proteins: Regulators of apoptosis and chemoresistance in hematologic malignancies. Semin. Hematol.

[2] Youle R.J., Strasser A. (2008). The BCL-2 protein family: Opposing activities that mediate cell death. Nat. Rev. Mol. Cell Biol.

[3] Reed J.C. (1994). Bcl-2 and the regulation of programmed cell death. J. Cell Biol.

[4] Salvesen G.S., Abrams J.M. (2004). Caspase activation—Stepping on the gas or releasing the brakes? Lessons from humans and flies. Oncogene.

[5] Salvesen G.S., Duckett C.S. (2002). IAP proteins: Blocking the road to death’s door. Nat. Rev. Mol. Cell Biol.

[6] Hawkins C.J., Silke J., Verhagen A.M., Foster R., Ekert P.G., Ashley D.M. (2001). Analysis of candidate antagonists of IAP-mediated caspase inhibition using yeast reconstituted with the mammalian Apaf-1-activated apoptosis mechanism. Apoptosis.

[7] Hofmann K., Bucher P., Tschopp J. (1997). The CARD domain: A new apoptotic signalling motif. Trends Biochem. Sci.

[8] Eckelman B.P., Salvesen G.S., Scott F.L. (2006). Human inhibitor of apoptosis proteins: Why XIAP is the black sheep of the family. EMBO Rep.

[9] Suzuki Y., Nakabayashi Y., Takahashi R. (2001). Ubiquitin-protein ligase activity of X-linked inhibitor of apoptosis protein promotes proteasomal degradation of caspase-3 and enhances its anti-apoptotic effect in Fas-induced cell death. Proc. Natl. Acad. Sci. USA.

[10] Chai J., Du C., Wu J.W., Kyin S., Wang X., Shi Y. (2000). Structural and biochemical basis of apoptotic activation by Smac/DIABLO. Nature.

[11] Liu Z., Sun C., Olejniczak E.T., Meadows R.P., Betz S.F., Oost T., Herrmann J., Wu J.C., Fesik S.W. (2000). Structural basis for binding of Smac/DIABLO to the XIAP BIR3 domain. Nature.

[12] Srinivasula S.M., Datta P., Fan X.J., Fernandes-Alnemri T., Huang Z., Alnemri E.S. (2000). Molecular determinants of the caspase-promoting activity of Smac/DIABLO and its role in the death receptor pathway. J. Biol. Chem.

[13] Srinivasula S.M., Hegde R., Saleh A., Datta P., Shiozaki E., Chai J., Lee R.A., Robbins P.D., Fernandes-Alnemri T., Shi Y., Alnemri E.S. (2001). A conserved XIAP-interaction motif in caspase-9 and Smac/DIABLO regulates caspase activity and apoptosis. Nature.

[14] Wu G., Chai J., Suber T.L., Wu J.W., Du C., Wang X., Shi Y. (2000). Structural basis of IAP recognition by Smac/DIABLO. Nature.

[15] Fraser A.G., James C., Evan G.I., Hengartner M.O. (1999). Caenorhabditis elegans inhibitor of apoptosis protein (IAP) homologue BIR-1 plays a conserved role in cytokinesis. Curr. Biol.

[16] Uren A.G., Beilharz T., O’Connell M.J., Bugg S.J., van Driel R., Vaux D.L., Lithgow T. (1999). Role for yeast inhibitor of apoptosis (IAP)-like proteins in cell division. Proc. Natl. Acad. Sci. USA.

[17] Speliotes E.K., Uren A., Vaux D., Horvitz H.R. (2000). The survivin-like *C. elegans* BIR-1 protein acts with the Aurora-like kinase AIR-2 to affect chromosomes and the spindle midzone. Mol. Cell.

[18] Li F., Ambrosini G., Chu E.Y., Plescia J., Tognin S., Marchisio P.C., Altieri D.C. (1998). Control of apoptosis and mitotic spindle checkpoint by survivin. Nature.

[19] Uren A.G., Wong L., Pakusch M., Fowler K.J., Burrows F.J., Vaux D.L., Choo K.H. (2000). Survivin and the inner centromere protein INCENP show similar cell-cycle localization and gene knockout phenotype. Curr. Biol.

[20] Hanahan D., Weinberg R.A. (2000). The hallmarks of cancer. Cell.

[21] Tamm I., Richter S., Scholz F., Schmelz K., Oltersdorf D., Karawajew L., Schoch C., Haferlach T., Ludwig W.D., Wuchter C. (2004). XIAP expression correlates with monocytic differentiation in adult *de novo* AML: Impact on prognosis. Hematol. J.

[22] Yang L., Cao Z., Yan H., Wood W.C. (2003). Coexistence of high levels of apoptotic signaling and inhibitor of apoptosis proteins in human tumor cells: Implication for cancer specific therapy. Cancer Res.

[23] Tamm I., Kornblau S.M., Segall H., Krajewski S., Welsh K., Kitada S., Scudiero D.A., Tudor G., Qui Y.H., Monks A. (2000). Expression and prognostic significance of IAP-family genes in human cancers and myeloid leukemias. Clin. Cancer Res.

[24] Nachmias B., Ashhab Y., Ben-Yehuda D. (2004). The inhibitor of apoptosis protein family (IAPs): An emerging therapeutic target in cancer. Semin. Cancer Biol.

[25] Schimmer A.D. (2004). Inhibitor of apoptosis proteins: Translating basic knowledge into clinical practice. Cancer Res.

[26] Wright C.W., Duckett C.S. (2005). Reawakening the cellular death program in neoplasia through the therapeutic blockade of IAP function. J. Clin. Invest.

[27] Xu R., Zhang P., Huang J., Ge S., Lu J., Qian G. (2007). Sp1 and Sp3 regulate basal transcription of the survivin gene. Biochem. Biophys. Res. Commun.

[28] Jiang Y., Saavedra H.I., Holloway M.P., Leone G., Altura R.A. (2004). Aberrant regulation of survivin by the RB/E2F family of proteins. J. Biol Chem.

[29] Hoffman W.H., Biade S., Zilfou J.T., Chen J., Murphy M. (2002). Transcriptional repression of the anti-apoptotic survivin gene by wild type p53. J. Biol. Chem.

[30] Meng R.D., Shelton C.C., Li Y.M., Qin L.X., Notterman D., Paty P.B., Schwartz G.K. (2009). gamma-Secretase inhibitors abrogate oxaliplatin-induced activation of the Notch-1 signaling pathway in colon cancer cells resulting in enhanced chemosensitivity. Cancer Res.

[31] Chen Y., Li D., Liu H., Xu H., Zheng H., Qian F., Li W., Zhao C., Wang Z., Wang X. (2011). Notch-1 signaling facilitates survivin expression in human non-small cell lung cancer cells. Cancer Biol. Ther.

[32] Vaira V., Lee C.W., Goel H.L., Bosari S., Languino L.R., Altieri D.C. (2007). Regulation of survivin expression by IGF-1/mTOR signaling. Oncogene.

[33] Fortugno P., Beltrami E., Plescia J., Fontana J., Pradhan D., Marchisio P.C., Sessa W.C., Altieri D.C. (2003). Regulation of survivin function by Hsp90. Proc. Natl. Acad. Sci. USA.

[34] Wang H., Gambosova K., Cooper Z.A., Holloway M.P., Kassai A., Izquierdo D., Cleveland K., Boney C.M., Altura R.A. (2010). EGF regulates survivin stability through the Raf-1/ERK pathway in insulin-secreting pancreatic beta-cells. BMC Mol. Biol.

[35] Ju J.H., Yang W., Oh S., Nam K., Lee K.M., Noh D.Y., Shin I. (2013). HER2 stabilizes survivin while concomitantly down-regulating survivin gene transcription by suppressing Notch cleavage. Biochem. J.

[36] Siddiqa A., Long L.M., Li L., Marciniak R.A., Kazhdan I. (2008). Expression of HER-2 in MCF-7 breast cancer cells modulates anti-apoptotic proteins Survivin and Bcl-2 via the extracellular signal-related kinase (ERK) and phosphoinositide-3 kinase (PI3K) signalling pathways. BMC Cancer.

[37] Beierle E.A., Nagaram A., Dai W., Iyengar M., Chen M.K. (2005). VEGF-mediated survivin expression in neuroblastoma cells. J. Surg. Res.

[38] O’Connor D.S., Grossman D., Plescia J., Li F., Zhang H., Villa A., Tognin S., Marchisio P.C., Altieri D.C. (2000). Regulation of apoptosis at cell division by p34cdc2 phosphorylation of survivin. Proc. Natl. Acad. Sci. USA.

[39] Zhou S., Li L., Jian X., Ou X., Jiang H., Yao Z., Xu C., Peng J. (2008). The phosphorylation of survivin Thr34 by p34cdc2 in carcinogenesis of oral submucous fibrosis. Oncol. Rep.

[40] Pannone G., Bufo P., Serpico R., Rubini C., Zamparese R., Corsi F., Pedicillo M.C., Staibano S., de Rosa G., Lo Muzio L. (2007). Survivin phosphorylation and M-phase promoting factor in oral carcinogenesis. Histol. Histopathol.

[41] Dohi T., Beltrami E., Wall N.R., Plescia J., Altieri D.C. (2004). Mitochondrial survivin inhibits apoptosis and promotes tumorigenesis. J. Clin. Invest.

[42] Dohi T., Xia F., Altieri D.C. (2007). Compartmentalized phosphorylation of IAP by protein kinase A regulates cytoprotection. Mol. Cell.

[43] Wang H., Holloway M.P., Ma L., Cooper Z.A., Riolo M., Samkari A., Elenitoba-Johnson K.S., Chin Y.E., Altura R.A. (2010). Acetylation directs survivin nuclear localization to repress STAT3 oncogenic activity. J. Biol. Chem.

[44] Fukuda S., Pelus L.M. (2006). Survivin, a cancer target with an emerging role in normal adult tissues. Mol. Cancer Ther.

[45] Xing Z., Conway E.M., Kang C., Winoto A. (2004). Essential role of survivin, an inhibitor of apoptosis protein, in T cell development, maturation, and homeostasis. J. Exp. Med.

[46] Fukuda S., Pelus L.M. (2001). Regulation of the inhibitor-of-apoptosis family member survivin in normal cord blood and bone marrow CD34(+) cells by hematopoietic growth factors: Implication of survivin expression in normal hematopoiesis. Blood.

[47] Mesri M., Morales-Ruiz M., Ackermann E.J., Bennett C.F., Pober J.S., Sessa W.C., Altieri D.C. (2001). Suppression of vascular endothelial growth factor-mediated endothelial cell protection by survivin targeting. Am. J. Pathol.

[48] Deguchi M., Shiraki K., Inoue H., Okano H., Ito T., Yamanaka T., Sugimoto K., Sakai T., Ohmori S., Murata K. (2002). Expression of survivin during liver regeneration. Biochem. Biophys. Res. Commun.

[49] Chiou S.K., Moon W.S., Jones M.K., Tarnawski A.S. (2003). Survivin expression in the stomach: Implications for mucosal integrity and protection. Biochem. Biophys. Res. Commun.

[50] Gurbuxani S., Xu Y., Keerthivasan G., Wickrema A., Crispino J.D. (2005). Differential requirements for survivin in hematopoietic cell development. Proc. Natl. Acad. Sci. USA.

[51] Altznauer F., Martinelli S., Yousefi S., Thurig C., Schmid I., Conway E.M., Schoni M.H., Vogt P., Mueller C., Fey M.F. (2004). Inflammation-associated cell cycle-independent block of apoptosis by survivin in terminally differentiated neutrophils. J. Exp. Med.

[52] Altieri D.C. (2003). Survivin, versatile modulation of cell division and apoptosis in cancer. Oncogene.

[53] Adida C., Berrebi D., Peuchmaur M., Reyes-Mugica M., Altieri D.C. (1998). Anti-apoptosis gene, survivin, and prognosis of neuroblastoma. Lancet.

[54] Kawasaki H., Altieri D.C., Lu C.D., Toyoda M., Tenjo T., Tanigawa N. (1998). Inhibition of apoptosis by survivin predicts shorter survival rates in colorectal cancer. Cancer Res.

[55] Ambrosini G., Adida C., Altieri D.C. (1997). A novel anti-apoptosis gene, survivin, expressed in cancer and lymphoma. Nat. Med.

[56] Lu C.D., Altieri D.C., Tanigawa N. (1998). Expression of a novel antiapoptosis gene, survivin, correlated with tumor cell apoptosis and p53 accumulation in gastric carcinomas. Cancer Res.

[57] Grossman D., Kim P.J., Blanc-Brude O.P., Brash D.E., Tognin S., Marchisio P.C., Altieri D.C. (2001). Transgenic expression of survivin in keratinocytes counteracts UVB-induced apoptosis and cooperates with loss of p53. J. Clin Invest.

[58] Yamamoto T., Manome Y., Nakamura M., Tanigawa N. (2002). Downregulation of survivin expression by induction of the effector cell protease receptor-1 reduces tumor growth potential and results in an increased sensitivity to anticancer agents in human colon cancer. Eur J. Cancer.

[59] Mirza A., McGuirk M., Hockenberry T.N., Wu Q., Ashar H., Black S., Wen S.F., Wang L., Kirschmeier P., Bishop W.R. (2002). Human survivin is negatively regulated by wild-type p53 and participates in p53-dependent apoptotic pathway. Oncogene.

[60] Suzuki A., Ito T., Kawano H., Hayashida M., Hayasaki Y., Tsutomi Y., Akahane K., Nakano T., Miura M., Shiraki K. (2000). Survivin initiates procaspase 3/p21 complex formation as a result of interaction with Cdk4 to resist Fas-mediated cell death. Oncogene.

[61] Azuhata T., Scott D., Griffith T.S., Miller M., Sandler A.D. (2006). Survivin inhibits apoptosis induced by TRAIL, and the ratio between survivin and TRAIL receptors is predictive of recurrent disease in neuroblastoma. J. Pediatr. Surg.

[62] Tamm I., Wang Y., Sausville E., Scudiero D.A., Vigna N., Oltersdorf T., Reed J.C. (1998). IAP-family protein survivin inhibits caspase activity and apoptosis induced by Fas (CD95), Bax, caspases, and anticancer drugs. Cancer Res.

[63] Kobayashi K., Hatano M., Otaki M., Ogasawara T., Tokuhisa T. (1999). Expression of a murine homologue of the inhibitor of apoptosis protein is related to cell proliferation. Proc. Natl. Acad. Sci. USA.

[64] Shin S., Sung B.J., Cho Y.S., Kim H.J., Ha N.C., Hwang J.I., Chung C.W., Jung Y.K., Oh B.H. (2001). An anti-apoptotic protein human survivin is a direct inhibitor of caspase-3 and -7. Biochemistry.

[65] Conway E.M., Pollefeyt S., Steiner-Mosonyi M., Luo W., Devriese A., Lupu F., Bono F., Leducq N., Dol F., Schaeffer P. (2002). Deficiency of survivin in transgenic mice exacerbates Fas-induced apoptosis via mitochondrial pathways. Gastroenterology.

[66] Banks D.P., Plescia J., Altieri D.C., Chen J., Rosenberg S.H., Zhang H., Ng S.C. (2000). Survivin does not inhibit caspase-3 activity. Blood.

[67] Vagnarelli P., Earnshaw W.C. (2004). Chromosomal passengers: The four-dimensional regulation of mitotic events. Chromosoma.

[68] Jeyaprakash A.A., Klein U.R., Lindner D., Ebert J., Nigg E.A., Conti E. (2007). Structure of a Survivin-Borealin-INCENP core complex reveals how chromosomal passengers travel together. Cell.

[69] Ghosh J.C., Dohi T., Raskett C.M., Kowalik T.F., Altieri D.C. (2006). Activated checkpoint kinase 2 provides a survival signal for tumor cells. Cancer Res.

[70] Altieri D.C. (2006). The case for survivin as a regulator of microtubule dynamics and cell-death decisions. Curr. Opin. Cell Biol.

[71] Giodini A., Kallio M.J., Wall N.R., Gorbsky G.J., Tognin S., Marchisio P.C., Symons M., Altieri D.C. (2002). Regulation of microtubule stability and mitotic progression by survivin. Cancer Res.

[72] Zaffaroni N., Pennati M., Colella G., Perego P., Supino R., Gatti L., Pilotti S., Zunino F., Daidone M.G. (2002). Expression of the anti-apoptotic gene survivin correlates with taxol resistance in human ovarian cancer. Cell. Mol. Life Sci.

[73] Zhang M., Mukherjee N., Bermudez R.S., Latham D.E., Delaney M.A., Zietman A.L., Shipley W.U., Chakravarti A. (2005). Adenovirus-mediated inhibition of survivin expression sensitizes human prostate cancer cells to paclitaxel *in vitro* and *in vivo*. Prostate.

[74] O’Connor D.S., Wall N.R., Porter A.C., Altieri D.C. (2002). A p34(cdc2) survival checkpoint in cancer. Cancer Cell.

[75] Cong X.L., Han Z.C. (2004). Survivin and leukemia. Int. J. Hematol.

[76] Van Geelen C.M., de Vries E.G., de Jong S. (2004). Lessons from TRAIL-resistance mechanisms in colorectal cancer cells: Paving the road to patient-tailored therapy. Drug Resist. Updat.

[77] Nomura T., Yamasaki M., Nomura Y., Mimata H. (2005). Expression of the inhibitors of apoptosis proteins in cisplatin-resistant prostate cancer cells. Oncol. Rep.

[78] Tirro E., Consoli M.L., Massimino M., Manzella L., Frasca F., Sciacca L., Vicari L., Stassi G., Messina L., Messina A. (2006). Altered expression of c-IAP1, survivin, and Smac contributes to chemotherapy resistance in thyroid cancer cells. Cancer Res.

[79] Zhang M., Latham D.E., Delaney M.A., Chakravarti A. (2005). Survivin mediates resistance to antiandrogen therapy in prostate cancer. Oncogene.

[80] Asanuma K., Moriai R., Yajima T., Yagihashi A., Yamada M., Kobayashi D., Watanabe N. (2000). Survivin as a radioresistance factor in pancreatic cancer. Jpn J. Cancer Res.

[81] Pennati M., Binda M., Colella G., Folini M., Citti L., Villa R., Daidone M.G., Zaffaroni N. (2003). Radiosensitization of human melanoma cells by ribozyme-mediated inhibition of survivin expression. J. Invest. Dermatol.

[82] Rodel C., Haas J., Groth A., Grabenbauer G.G., Sauer R., Rodel F. (2003). Spontaneous and radiation-induced apoptosis in colorectal carcinoma cells with different intrinsic radiosensitivities: Survivin as a radioresistance factor. Int. J. Radiat. Oncol. Biol. Phys.

[83] Lo Muzio L., Pannone G., Leonardi R., Staibano S., Mignogna M.D., de Rosa G., Kudo Y., Takata T., Altieri D.C. (2003). Survivin, a potential early predictor of tumor progression in the oral mucosa. J. Dent. Res.

[84] Ye C.P., Qiu C.Z., Huang Z.X., Su Q.C., Zhuang W., Wu R.L., Li X.F. (2007). Relationship between survivin expression and recurrence, and prognosis in hepatocellular carcinoma. World J. Gastroenterol.

[85] Rosato A., Pivetta M., Parenti A., Iaderosa G.A., Zoso A., Milan G., Mandruzzato S., Del Bianco P., Ruol A., Zaninotto G. (2006). Survivin in esophageal cancer: An accurate prognostic marker for squamous cell carcinoma but not adenocarcinoma. Int. J. Cancer.

[86] Shirai K., Suzuki Y., Oka K., Noda S.E., Katoh H., Itoh J., Itoh H., Ishiuchi S., Sakurai H., Hasegawa M. (2009). Nuclear survivin expression predicts poorer prognosis in glioblastoma. J. Neurooncol.

[87] Mohamed S., Yasufuku K., Nakajima T., Hiroshima K., Chiyo M., Yoshida S., Suzuki M., Sekine Y., Shibuya K., Agamy G. (2009). Nuclear survivin in pN2 nonsmall cell lung cancer: Prognostic and clinical implications. Eur. Respir. J.

[88] Brennan D.J., Rexhepaj E., O’Brien S.L., McSherry E., O’Connor D.P., Fagan A., Culhane A.C., Higgins D.G., Jirstrom K., Millikan R.C. (2008). Altered cytoplasmic-to-nuclear ratio of survivin is a prognostic indicator in breast cancer. Clin. Cancer Res.

[89] Yamashita S., Masuda Y., Kurizaki T., Haga Y., Murayama T., Ikei S., Kamei M., Takeno S., Kawahara K. (2007). Survivin expression predicts early recurrence in early-stage breast cancer. Anticancer Res.

[90] Fan J., Wang L., Jiang G.N., He W.X., Ding J.A. (2008). The role of survivin on overall survival of non-small cell lung cancer, a meta-analysis of published literatures. Lung Cancer.

[91] Grossman D., McNiff J.M., Li F., Altieri D.C. (1999). Expression and targeting of the apoptosis inhibitor, survivin, in human melanoma. J. Invest. Dermatol.

[92] Cao C., Mu Y., Hallahan D.E., Lu B. (2004). XIAP and survivin as therapeutic targets for radiation sensitization in preclinical models of lung cancer. Oncogene.

[93] Sharma H., Sen S., Lo Muzio L., Mariggio A., Singh N. (2005). Antisense-mediated downregulation of anti-apoptotic proteins induces apoptosis and sensitizes head and neck squamous cell carcinoma cells to chemotherapy. Cancer Biol. Ther.

[94] Du Z.X., Zhang H.Y., Gao da X., Wang H.Q., Li Y.J., Liu G.L. (2006). Antisurvivin oligonucleotides inhibit growth and induce apoptosis in human medullary thyroid carcinoma cells. Exp. Mol. Med.

[95] Fuessel S., Kueppers B., Ning S., Kotzsch M., Kraemer K., Schmidt U., Meye A., Wirth M.P. (2004). Systematic *in vitro* evaluation of survivin directed antisense oligodeoxynucleotides in bladder cancer cells. J. Urol.

[96] Ansell S.M., Arendt B.K., Grote D.M., Jelinek D.F., Novak A.J., Wellik L.E., Remstein E.D., Bennett C.F., Fielding A. (2004). Inhibition of survivin expression suppresses the growth of aggressive non-Hodgkin’s lymphoma. Leukemia.

[97] Fisker N., Westergaard M., Hansen H.F., Hansen J.B. (2007). Survivin mRNA antagonists using locked nucleic acid, potential for molecular cancer therapy. Nucleosides Nucleotides Nucleic Acids.

[98] Carter B.Z., Mak D.H., Schober W.D., Cabreira-Hansen M., Beran M., McQueen T., Chen W., Andreeff M. (2006). Regulation of survivin expression through Bcr-Abl/MAPK cascade: Targeting survivin overcomes imatinib resistance and increases imatinib sensitivity in imatinib-responsive CML cells. Blood.

[99] Hayashi N., Asano K., Suzuki H., Yamamoto T., Tanigawa N., Egawa S., Manome Y. (2005). Adenoviral infection of survivin antisense sensitizes prostate cancer cells to etoposide *in vivo*. Prostate.

[100] Olie R.A., Simoes-Wust A.P., Baumann B., Leech S.H., Fabbro D., Stahel R.A., Zangemeister-Wittke U. (2000). A novel antisense oligonucleotide targeting survivin expression induces apoptosis and sensitizes lung cancer cells to chemotherapy. Cancer Res.

[101] Sah N.K., Munshi A., Hobbs M., Carter B.Z., Andreeff M., Meyn R.E. (2006). Effect of downregulation of survivin expression on radiosensitivity of human epidermoid carcinoma cells. Int. J. Radiat. Oncol. Biol. Phys.

[102] Kanwar J.R., Shen W.P., Kanwar R.K., Berg R.W., Krissansen G.W. (2001). Effects of survivin antagonists on growth of established tumors and B7-1 immunogene therapy. J. Natl. Cancer Inst.

[103] Pennati M., Colella G., Folini M., Citti L., Daidone M.G., Zaffaroni N. (2002). Ribozyme-mediated attenuation of survivin expression sensitizes human melanoma cells to cisplatin-induced apoptosis. J. Clin. Invest.

[104] Pennati M., Binda M., de Cesare M., Pratesi G., Folini M., Citti L., Daidone M.G., Zunino F., Zaffaroni N. (2004). Ribozyme-mediated down-regulation of survivin expression sensitizes human melanoma cells to topotecan *in vitro* and *in vivo*. Carcinogenesis.

[105] Carvalho A., Carmena M., Sambade C., Earnshaw W.C., Wheatley S.P. (2003). Survivin is required for stable checkpoint activation in taxol-treated HeLa cells. J. Cell Sci.

[106] Ling X., Li F. (2004). Silencing of antiapoptotic survivin gene by multiple approaches of RNA interference technology. Biotechniques.

[107] Ai Z., Yin L., Zhou X., Zhu Y., Zhu D., Yu Y., Feng Y. (2006). Inhibition of survivin reduces cell proliferation and induces apoptosis in human endometrial cancer. Cancer.

[108] Paduano F., Villa R., Pennati M., Folini M., Binda M., Daidone M.G., Zaffaroni N. (2006). Silencing of survivin gene by small interfering RNAs produces supra-additive growth suppression in combination with 17-allylamino-17-demethoxygeldanamycin in human prostate cancer cells. Mol. Cancer Ther.

[109] Nakao K., Hamasaki K., Ichikawa T., Arima K., Eguchi K., Ishii N. (2006). Survivin downregulation by siRNA sensitizes human hepatoma cells to TRAIL-induced apoptosis. Oncol. Rep.

[110] Lens S.M., Wolthuis R.M., Klompmaker R., Kauw J., Agami R., Brummelkamp T., Kops G., Medema R.H. (2003). Survivin is required for a sustained spindle checkpoint arrest in response to lack of tension. EMBO J.

[111] Beltrami E., Plescia J., Wilkinson J.C., Duckett C.S., Altieri D.C. (2004). Acute ablation of survivin uncovers p53-dependent mitotic checkpoint functions and control of mitochondrial apoptosis. J. Biol. Chem.

[112] Song H., Xin X.Y., Xiao F., Wang D.T., Yue Q.H., Han X. (2008). Survivin gene RNA interference inhibits proliferation, induces apoptosis, and enhances radiosensitivity in HeLa cells. Eur. J. Obstet. Gynecol. Reprod. Biol.

[113] Huynh T., Walchli S., Sioud M. (2006). Transcriptional targeting of small interfering RNAs into cancer cells. Biochem. Biophys. Res. Commun.

[114] Kappler M., Taubert H., Bartel F., Blumke K., Panian M., Schmidt H., Dunst J., Bache M. (2005). Radiosensitization, after a combined treatment of survivin siRNA and irradiation, is correlated with the activation of caspases 3 and 7 in a wt-p53 sarcoma cell line, but not in a mt-p53 sarcoma cell line. Oncol. Rep.

[115] Yonesaka K., Tamura K., Kurata T., Satoh T., Ikeda M., Fukuoka M., Nakagawa K. (2006). Small interfering RNA targeting survivin sensitizes lung cancer cell with mutant p53 to adriamycin. Int. J. Cancer.

[116] Jiang G., Li J., Zeng Z., Xian L. (2006). Lentivirus-mediated gene therapy by suppressing survivin in BALB/c nude mice bearing oral squamous cell carcinoma. Cancer Biol. Ther.

[117] Plescia J., Salz W., Xia F., Pennati M., Zaffaroni N., Daidone M.G., Meli M., Dohi T., Fortugno P., Nefedova Y. (2005). Rational design of shepherdin, a novel anticancer agent. Cancer Cell.

[118] Pennati M., Campbell A.J., Curto M., Binda M., Cheng Y., Wang L.Z., Curtin N., Golding B.T., Griffin R.J., Hardcastle I.R. (2005). Potentiation of paclitaxel-induced apoptosis by the novel cyclin-dependent kinase inhibitor NU6140: A possible role for survivin down-regulation. Mol. Cancer Ther.

[119] Nakahara T., Takeuchi M., Kinoyama I., Minematsu T., Shirasuna K., Matsuhisa A., Kita A., Tominaga F., Yamanaka K., Kudoh M. (2007). YM155, a novel small-molecule survivin suppressant, induces regression of established human hormone-refractory prostate tumor xenografts. Cancer Res.

[120] Chang C.C., Heller J.D., Kuo J., Huang R.C. (2004). Tetra-*O*-methyl nordihydroguaiaretic acid induces growth arrest and cellular apoptosis by inhibiting Cdc2 and survivin expression. Proc. Natl. Acad. Sci. USA.

[121] Smolewski P. (2008). Terameprocol, a novel site-specific transcription inhibitor with anticancer activity. IDrugs.

[122] Xiang R., Mizutani N., Luo Y., Chiodoni C., Zhou H., Mizutani M., Ba Y., Becker J.C., Reisfeld R.A. (2005). A DNA vaccine targeting survivin combines apoptosis with suppression of angiogenesis in lung tumor eradication. Cancer Res.

[123] Zhu K., Qin H., Cha S.C., Neelapu S.S., Overwijk W., Lizee G.A., Abbruzzese J.L., Hwu P., Radvanyi L., Kwak L.W. (2007). Survivin DNA vaccine generated specific antitumor effects in pancreatic carcinoma and lymphoma mouse models. Vaccine.

[124] Fuessel S., Meye A., Schmitz M., Zastrow S., Linne C., Richter K., Lobel B., Hakenberg O.W., Hoelig K., Rieber E.P. (2006). Vaccination of hormone-refractory prostate cancer patients with peptide cocktail-loaded dendritic cells: Results of a phase I clinical trial. Prostate.

[125] Tu S.P., Jiang X.H., Lin M.C., Cui J.T., Yang Y., Lum C.T., Zou B., Zhu Y.B., Jiang S.H., Wong W.M. (2003). Suppression of survivin expression inhibits *in vivo* tumorigenicity and angiogenesis in gastric cancer. Cancer Res.

[126] Mesri M., Wall N.R., Li J., Kim R.W., Altieri D.C. (2001). Cancer gene therapy using a survivin mutant adenovirus. J. Clin. Invest.

[127] Grossman D., Kim P.J., Schechner J.S., Altieri D.C. (2001). Inhibition of melanoma tumor growth *in vivo* by survivin targeting. Proc. Natl. Acad. Sci. USA.

[128] Blanc-Brude O.P., Mesri M., Wall N.R., Plescia J., Dohi T., Altieri D.C. (2003). Therapeutic targeting of the survivin pathway in cancer: Initiation of mitochondrial apoptosis and suppression of tumor-associated angiogenesis. Clin. Cancer Res.

[129] Gaj T., Gersbach C.A., Barbas C.F. (2013). ZFN, TALEN, and CRISPR/Cas-based methods for genome engineering. Trends Biotechnol.

[130] Carroll D. (2011). Genome engineering with zinc-finger nucleases. Genetics.

[131] Christian M., Cermak T., Doyle E.L., Schmidt C., Zhang F., Hummel A., Bogdanove A.J., Voytas D.F. (2010). Targeting DNA double-strand breaks with TAL effector nucleases. Genetics.

[132] Voll R.E., Herrmann M., Roth E.A., Stach C., Kalden J.R., Girkontaite I. (1997). Immunosuppressive effects of apoptotic cells. Nature.

[133] Fadok V.A., Bratton D.L., Konowal A., Freed P.W., Westcott J.Y., Henson P.M. (1998). Macrophages that have ingested apoptotic cells *in vitro* inhibit proinflammatory cytokine production through autocrine/paracrine mechanisms involving TGF-beta, PGE2, and PAF. J. Clin. Invest.

[134] McDonald P.P., Fadok V.A., Bratton D., Henson P.M. (1999). Transcriptional and translational regulation of inflammatory mediator production by endogenous TGF-beta in macrophages that have ingested apoptotic cells. J. Immunol.

[135] Fadok V.A., Bratton D.L., Guthrie L., Henson P.M. (2001). Differential effects of apoptotic *versus* lysed cells on macrophage production of cytokines: Role of proteases. J. Immunol.

[136] Morimoto K., Amano H., Sonoda F., Baba M., Senba M., Yoshimine H., Yamamoto H., Ii T., Oishi K., Nagatake T. (2001). Alveolar macrophages that phagocytose apoptotic neutrophils produce hepatocyte growth factor during bacterial pneumonia in mice. Am. J. Respir Cell Mol. Biol.

[137] Hristov M., Erl W., Linder S., Weber P.C. (2004). Apoptotic bodies from endothelial cells enhance the number and initiate the differentiation of human endothelial progenitor cells *in vitro*. Blood.

[138] Golpon H.A., Fadok V.A., Taraseviciene-Stewart L., Scerbavicius R., Sauer C., Welte T., Henson P.M., Voelkel N.F. (2004). Life after corpse engulfment: Phagocytosis of apoptotic cells leads to VEGF secretion and cell growth. FASEB J.

[139] White G.E., Tan T.C., John A.E., Whatling C., McPheat W.L., Greaves D.R. (2010). Fractalkine has anti-apoptotic and proliferative effects on human vascular smooth muscle cells via epidermal growth factor receptor signalling. Cardiovasc. Res.

[140] Hermens A.F., Barendsen G.W. (1969). Changes of cell proliferation characteristics in a rat rhabdomyosarcoma before and after x-irradiation. Eur. J. Cancer.

[141] Stephens T.C., Currie G.A., Peacock J.H. (1978). Repopulation of gamma-irradiated Lewis lung carcinoma by malignant cells and host macrophage progenitors. Br. J. Cancer.

[142] Huang Q., Li F., Liu X., Li W., Shi W., Liu F.F., O’Sullivan B., He Z., Peng Y., Tan A.C. (2011). Caspase 3-mediated stimulation of tumor cell repopulation during cancer radiotherapy. Nat. Med.

[143] Garg H., Salcedo R., Trinchieri G., Blumenthal R. (2010). Improved nonviral cancer suicide gene therapy using survivin promoter-driven mutant Bax. Cancer Gene Ther.

